# Klaus Minde MD FRCP(C): Formerly Chairman, Division of Child and Adolescent Psychiatry, McGill University, Montreal

**DOI:** 10.1192/pb.bp.116.055350

**Published:** 2017-02

**Authors:** Philip Graham

Klaus Minde, who recently died at the age of 82, was an academic child and adolescent psychiatrist renowned for his pioneering work in a variety of fields – especially infant psychiatry – and for the development of child mental health services in low-income countries. His early research was in the field of hyperactivity in children. Working with Gaby Weiss and John Werry, he was one of the first to carry out studies of medication in this condition.

In 1961, shortly after he had started his training in child psychiatry, his first child Thomas was born prematurely; he and his wife Nina, a psychologist, were not allowed to touch him – they could only gaze at him through the window of the nursery until they could take him home when he was 4 weeks old. This distressing experience stimulated Klaus' interest in the development of premature babies and the ways parents could be involved in their care. He carried out a number of observational studies^1^ which – allied with the development of ideas on mother-infant bonding promoted by two paediatricians, Marshall Klaus and John Kennell – helped to change practices in the nursing care of premature babies.

From 1971 he spent 2 years on secondment setting up a child psychiatric clinic at Makerere University, Uganda. As well as establishing a service, he carried out a significant amount of research, conducting comparative studies of disorders in Canada and Uganda.^2^ Idi Amin, the brutal, idiosyncratic president of the country, was in power at the time. Klaus described how Amin, as chancellor of the university, required every professor to be present when he had decided to address them. While everyone was waiting for him, 3 empty limousines would arrive, followed – 3 minutes later – by Amin on a bicycle that he had mounted just before entering the university.

In 1983 he spent a sabbatical year in London, where he met John Bowlby, whose work on attachment had so impressed him. He was able to persuade Bowlby to visit Canada to talk about his ideas. After his official retirement from his chairmanship in 2000, he and his wife spent a year in Johannesburg, South Africa, where they worked with very disadvantaged children and families in the townships of Alexandra and Soweto. Once again he carried out research, this time on the assessment of attachment.^3^ In addition, Nina and he organised a mutual support group of grandmothers looking after their grandchildren (orphaned by the country-wide epidemic of AIDS), which became a model for similar groups in the area.

Klaus always had a strong interest in the plight of disadvantaged children and their need for psychiatric services. From 1994 to 1999 he worked with native Canadian children, acting as a consultant to the Cree Board of Health in Mistissini, Quebec; from 2009 to 2015 he was a consultant to Dans La Rue, an organisation caring for street children in Montreal.

Klaus was born in Leipzig in 1933 in the same year as Hitler came to power in Germany. His father was technical director of Germany's public radio and strongly opposed the Nazi movement. As one of the few senior people who had refused to join the Nazi party, his father was promoted after the war but was later sacked when a communist state was imposed in Eastern Germany in 1949. Klaus attended a humanist school and also rebelled against the ruling party. He wanted to study medicine but given his family background and outspoken political views, he had no chance of entering an East German medical school. Leaving his family behind – it was many years before his mother could join him – he travelled to West Germany and obtained a place to read medicine in Munich. After qualification he won a Fulbright scholarship which enabled him to go to New York and gain experience in paediatrics at Bellevue Hospital and psychology at Columbia University. He then undertook residency training in psychiatry at McGill University in Montreal, remaining in Canada, except for his sabbatical periods, for the rest of his professional life. He was Director of Research at the Hospital for Sick Children, Toronto, from 1973 to 1986 and after a spell at Queen's University in Kingston, Ontario, he was appointed Chairman of the Division of Child and Adolescent Psychiatry at McGill University from 1989 to 2000.

With his boundless energy and enthusiasm, Klaus could set audiences alight with his rhetoric, promoting the cause of better care not just for newborn babies but for children with physical and mental disabilities and for the socially disadvantaged. His whole body, especially his gesticulating arms and plentiful hair, seemed to be engaged in getting over his messages to his audiences, who loved it!

After retirement he continued to mentor colleagues and see patients until he suffered a major stroke shortly before his death on 6 July 2016. He is survived by Nina, his three sons and four grandchildren.
